# Non-coding RNAs in the spotlight of the pathogenesis, diagnosis, and therapy of cutaneous T cell lymphoma

**DOI:** 10.1038/s41420-024-02165-2

**Published:** 2024-09-10

**Authors:** Xiao He, Qian Zhang, Yimeng Wang, Jiachen Sun, Ying Zhang, Chunlei Zhang

**Affiliations:** Department of Dermatology, Peking University Third Hospital, Beijing, China

**Keywords:** Cancer genomics, Cancer microenvironment, Cancer therapeutic resistance, Tumour biomarkers

## Abstract

Cutaneous T-cell lymphoma (CTCL) is a group of primary and secondary cutaneous malignancies characterized by aberrant T-cells in the skin. Diagnosing CTCL in its early stage can be difficult because of CTCL’s ability to mimic benign cutaneous inflammatory skin diseases. CTCL has multiple subtypes with different disease progression and diagnostic parameters despite similar clinical manifestations. The accurate diagnosis and prognosis of a varied range of diseases require the detection of molecular entities to capture the complete footprint of disease physiology. Non-coding RNAs (ncRNAs) have recently been discovered as major regulators of CTCL gene expression. They can affect tumor cell growth, migration, programmed cell death (PCD), and immunoregulation through interactions with the tumor microenvironment (TME), which in turn affect CTCL progression. This review summarizes recent advances in how ncRNAs regulate CTCL cell activity, especially their role in PCD. It also discusses the potential use of ncRNAs as diagnostic and prognostic biomarkers for different subtypes of CTCL. Furthermore, prospective targets and therapeutic approaches influenced by ncRNAs are presented. A better appreciation of the intricate epigenetic landscape of CTCL is expected to facilitate the creation of innovative targeted therapies for the condition.

## Facts

- NcRNAs have been the subject of considerable research interest, because of their regulatory activities in the flow of gene information in local and distal ecological niches.
- NcRNAs are correlated with the tumorigenesis, proliferation, metastasis, and PCD of CTCL.
- NcRNAs can be taken up non-randomly by both heterologous and homologous cells, influencing post-transcriptional genetic regulation and causing behavioral changes such as tumorigenesis, proliferation, metastasis, PCD, and immunological regulation.
- NcRNAs can be employed for the CTCL differential diagnosis, the classification of subtypes, and even the prediction of patient response to personalized treatment, which provides novel insights into the field of CTCL therapy.

## Open questions

- Whether the levels and abundance of ncRNAs change accordingly at different stages of CTCL progression?
- How can we accurately establish which ncRNA has the most influence on tumor progression at a certain stage and type of CTCL?
- Through which PCD do ncRNAs mainly affect CTCL progression?
- Which cell subpopulation do ncRNAs primarily act on when affecting CTCL immunomodulation?
- How do these ncRNAs influence therapeutic resistance in CTCL?

## Introduction

**Fig. 1:**
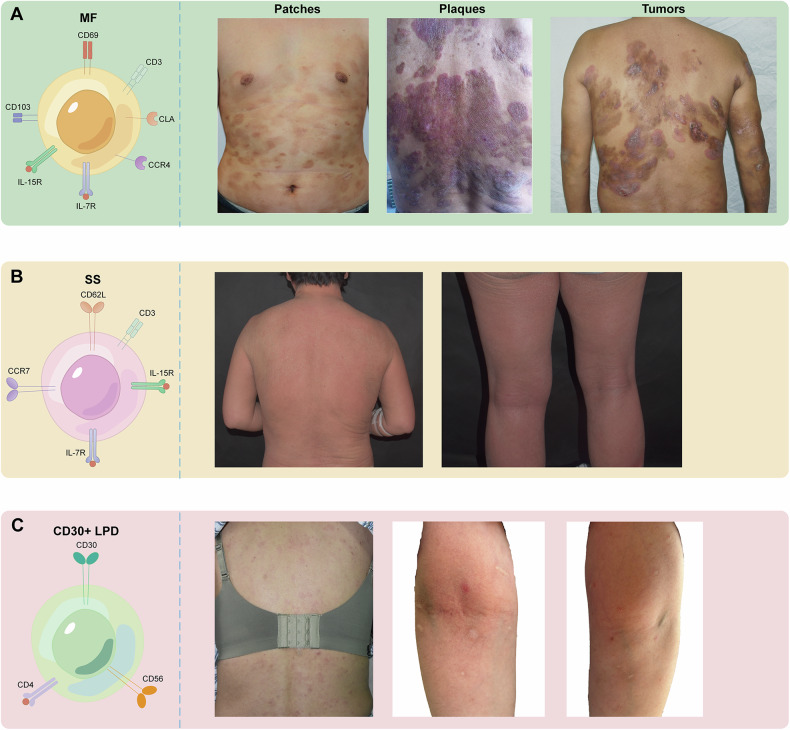
Common subtypes of CTCL and their stages. The three most prevalent subtypes of CTCL are MF, SS, and CD30 (+) LPD. **A** MF is divided into three main stages: patches, plaques, and tumors. In well-demarcated patch or plaque lesions of MF, malignant T cells usually exhibit the resident memory T cell phenotype with expression of CD69 and CD103. **B** SS presents as an erythema with diffuse infiltration of the skin, often accompanied by generalized lymph node enlargement, nail dystrophy, palmoplantar keratosis, and intense pruritus. Malignant T cells found in SS diffuse erythema typically have the central memory T cell phenotype with expression of CCR7 and CD62L. **C** LyP is characterized by erythematous to brown papules and small nodules less than 2 cm in size, preferentially on the trunk and extremities. Erythematous patches or larger non-resolving tumors in patients with LyP should raise suspicion of MF or PCALCL. The phenotype of CD30 (+) LPD is characterized by the CD30 antigen. CTCL cutaneous T-cell lymphoma, MF mycosis fungoides, SS Sézary syndrome, LPD lymphoproliferative disorder, LyP lymphomatoid papulosis, PCALCL primary cutaneous anaplastic large cell lymphoma.

Cutaneous T-cell lymphoma (CTCL) is a rare disease characterized by the accumulation of neoplastic lymphocytes in the skin, and the incidence of CTCL is 0.96 per 100,000 [1, 2]. The subtypes of CTCL are diverse in clinical manifestations and are defined according to the prognosis, histopathological features, and organ involvement [3]. The three most prevalent subtypes of CTCL are mycosis fungoides (MF), Sézary syndrome (SS), and CD30 (+) lymphoproliferative disorder (LPD), which constitute around 75-80% of all cases [3]. MF is the most prevalent type of CTCL. It is typically slow-growing and early-stage MF manifests as patches and plaques. However, the clinical course is protracted and marked by the transformation of isolated cutaneous lesions into infiltrating plaques or massive ulcerated tumors on preexisting plaques. Some individuals may develop to advanced stages, exhibiting cutaneous malignancies and/or malignant T cells with distant spread [4] (Fig. 1A). SS is a leukemic form of CTCL known for its rapid disease development, systemic involvement, and poor prognosis. It is distinguished by cutaneous involvement, which is marked by erythroderma with severe pruritus, as well as leukemic manifestations of tumor clones [5] (Fig. 1B). CD30 (+) LPD includes various diseases, such as lymphomatoid papulosis (LyP) and primary cutaneous anaplastic large cell lymphoma (PCALCL). These diseases have a recurring history and a favorable prognosis [6] (Fig. 1C). Early detection of CTCL is challenging since it resembles benign inflammatory dermatoses. In fact, diagnosis can take up to five years and require multiple skin biopsies. Despite recent research efforts, the pathophysiology of CTCL remains poorly understood.

The treatment of CTCL is primarily determined by the stage of the disease. Skin-directed treatment is the preferred approach for early-stage illness (stages IA-IIA), while systemic biological medications are necessary for patients with more widespread infiltration [7]. Systemic chemotherapy is typically reserved for patients with advanced or refractory/recurring CTCL [8]. Advanced CTCL is primarily treated palliatively, except for allogeneic stem cell transplantation, which is a curative treatment. However, the optimal regimen and timing for this therapy remain unknown. Although both single-agent and combination chemotherapy treatments have shown positive results, the overall outcomes are poorer compared to other lymphomas [9]. Therefore, there is an urgent need to discover effective therapies for patients with advanced CTCL.

**Fig. 2:**
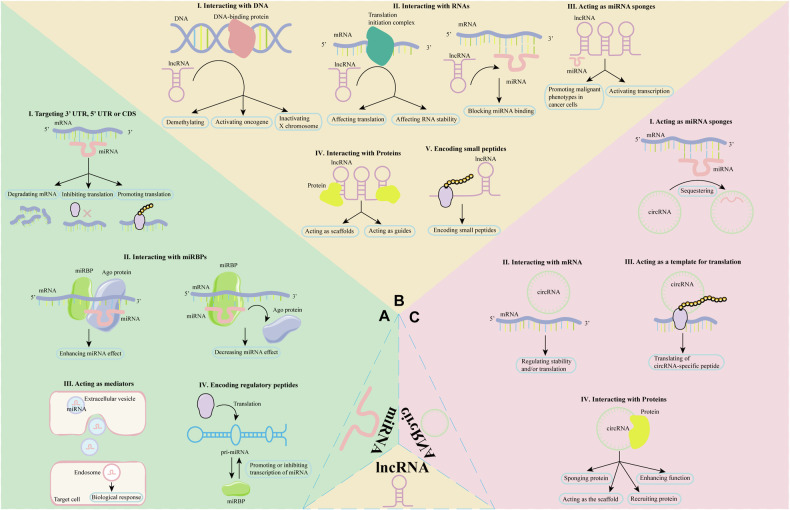
Functions of three major ncRNAs. **A** The classical function of miRNA is to target the 3’ UTR, 5’ UTR, or CDS of its target mRNA, resulting in mRNA degradation, translational repression, or activation. MiRNA can be secreted through extracellular vesicles and act as hormones and as mediators of intracellular communication. Some miRNAs can also interact with miRBPs, which can cooperate or compete with Ago proteins to enhance or silence the function of miRNAs on their target molecules. Some pri-miRNAs encode regulatory peptides that can influence the expression of mature miRNAs. **B** LncRNAs can bind directly to DNA to form R-loops or act as chromatin regulators in complexes with DNA-binding proteins. LncRNAs can interact with mRNAs to inhibit miRNAs by affecting translation, influencing RNA stability, or blocking miRNA binding sites. The sponging activity of lncRNAs is mainly through interaction with miRNAs. LncRNAs can interact with proteins and act as scaffolds or guides for them. Some lncRNAs have coding activity and produce micropeptides. **C** CircRNAs have the potential to be miRNA sponges. CircRNAs can interact with specific mRNAs and regulate their stability and/or translation. CircRNAs can be translated and produce small peptides. CircRNAs containing motifs that bind to RNA-binding proteins may act as decoys or sponges for proteins, thereby regulating their activity. CircRNAs containing motifs that promote the binding of enzymes to their substrates act as scaffolds, co-localizing the two molecules and optimizing reaction kinetics. CircRNAs can interact with gene promoters to enhance function. In addition, circRNAs can bind to the U1 snRNP and subsequently to the RNA polymerase II transcription complex, thereby increasing protein expression. NcRNAs non-coding RNAs, MiRNA microRNA, UTR untranslated region, CDS coding sequence, mRNA messenger RNA, MiRBP MiRNA-binding protein, Ago argonaute, LncRNA long non-coding RNA, CircRNA circular RNA, SnRNP small nuclear ribonucleoprotein.

Non-coding RNAs (ncRNAs) do not encode proteins and contribute to gene regulation, modification, and innate and adaptive immunity [10]. NcRNAs are classified into the following categories: microRNAs (miRNAs), long non-coding RNAs (lncRNAs), circular RNAs (circRNAs), heterogeneous nuclear RNAs (hnRNAs), PIWI-interacting RNAs (piRNAs), ribosomal RNAs (rRNAs), small nuclear RNAs (snRNAs), small nucleolar RNAs (snoRNAs), and transfer RNAs (tRNAs). Of these, the first three have been studied in more detail [11]. MiRNAs are small RNA molecules, usually 18-22 nucleotides long, that principally operate in post-transcriptional suppression of specific gene expression and RNA silencing [12]. They are the most well-studied type of ncRNA. An abnormal miRNA profile can promote the growth and invasion of CTCL (Fig. 2A). LncRNAs, on the other hand, are linear RNAs longer than 200 base pairs that cannot encode proteins but can affect gene expression [13]. Several lncRNAs, including TMEM244 and MALAT1, have been investigated for their involvement in the initiation and progression of CTCL [14] (Fig. 2B). CircRNAs are endogenous molecules that are diverse, evolutionarily conserved, relatively stable, and specific [15]. They typically function as miRNA sponges, regulating the transcription and splicing of their parental genes to either promote or repress tumors [16] (Fig. 2C). However, there have been few studies on the regulatory mechanism of circRNAs in CTCL. Therefore, this paper primarily focuses on lncRNAs and miRNAs.

Based on the characteristics outlined above, the presentation of ncRNAs in CTCL is complex and variable, making them critical for both diagnosis and treatment. This article provides a review of the latest research on the fundamental functions of ncRNAs in CTCL, including their role in CTCL behaviors, immunological regulation, diagnostic potential, and treatment options, as well as current research limitations and future perspectives. A comprehensive understanding of the ncRNA processes involved in CTCL formation can provide new insights into ncRNA-associated carcinogenesis and facilitate the development of innovative CTCL treatment techniques.

## NcRNAs in modulating CTCL behaviors

NcRNAs play a significant role in the communication between cells, which have a substantial influence on CTCL development [17]. They can be absorbed by heterologous and homologous cells in a nonrandom way, influencing post-transcriptional genetic regulation and causing behavioral changes such as tumor proliferation, metastasis, and programmed cell death (PCD) [18] (Table 1).

**Table 1:** Representative ncRNAs in CTCL and their related mechanisms.

NcRNAs	Dysregulation	Remodeling CTCL behaviors	Mechanism	Clinical significance
TMEM244	Upregulated in HH and SeAx cells	Promoted the growth of CTCL cells	Acted as a lncRNA that is necessary for the growth of CTCL cells	A new therapeutic target for the treatment of CTCL [14]
MALAT1	Upregulated in CTCL patients	Promoted the growth of CTCL cells	Induced EMT and cancer stem cell phenotype by sponging miR-124	A key step for CTCL targeting therapeutic benefit [19]
miR-106b	Upregulated in MF patients	Promoted the growth of MF tumor cells	Repressed expression of the tumor suppressors p21 and TXNIP	A potential future therapeutic target for MF [21]
miR-93	Upregulated in MF patients	Promoted the growth of malignant T cells in MF	Repressed expression of the cell cycle inhibitor p21	A treatment target for MF [22]
miR-135a	Downregulated in Hut78 cells	Inhibited the growth of CTCL cells	Inhibited the progress of CTCL via the GATA-3/TOX signaling pathway	A novel tumor suppressor for CTCL [23]
miR-223	Downregulated in early stage MF skin and HH and Hut78 cells	Inhibited the growth of CTCL cells	Repressed the expression of E2F1, MEF2C, and TOX	Being useful for the development of new therapeutics for MF/CTCL [24]
miR-17-5p	Downregulated in SS patients and SeAx cells	Inhibited the growth of SS cells	Increased apoptosis and decreased cell proliferation in SS cells	The new possibilities for the diagnosis and treatment of SS [25]
miR-195-5p	Downregulated in MF patients	Inhibited the growth of MF cells	Repressed the expression of ARL2	A future therapeutic target in MF [26]
miR-155	Upregulated in MF patients	Promoted the growth of CTCL cells	–	The first direct evidence that both malignant and non-malignant T cells express miR-155 in situ in CTCL [28]
miR-155	Upregulated in MyLa 和 MJ cells	Promoted the growth of MF cells	Interrupted activation of the G2/M checkpoint and decreased apoptosis	A novel therapeutic modalities for MF [30]
miR-155-5p/miR-130b-3p/miR-21-3p	Upregulated in CTCL patients, and MyLa and HuT78 cells	Promoted the growth of CTCL cells	Promoted the progress of CTCL via the IL6/JAK/STAT signaling pathway	The therapeutic targets in combination with immune checkpoint blockade to potentiate antitumor efficacy [33]
miR-155	Upregulated in CTCL patients and cells	Promoted the growth of CTCL cells	Promoted the progress of CTCL via the JAK/STAT, MAPK/ERK and PI3K/AKT signaling pathway	A way to assess pharmacodynamic response to cobomarsen therapy [34]
miR-155	Upregulated in CTCL patients and cells	Promoted the growth of CTCL cells	Promoted the progress of CTCL via the STAT5/BIC/miR-155 signaling pathway	A potential therapeutic target in CTCL [35]
MALAT1	Upregulated in MF patients	Promoted the metastasis of MF cells	Promoted the progress of MF via the activation of mTOR induced by CCL21	A potential therapeutic targets for MF [37]
miR-155/miR-1246	Upregulated in MyLa and MJ cells, and MF patients	Promoted the metastasis of MF cells and NPBMCs	–	The targets for novel treatments and promising noninvasive biomarkers for MF [38]
miR-26	Downregulated in MyLa and HH cells	Inhibited the metastasis of CTCL cells	Inhibited the metastasis of CTCL via the IL-22-STAT3-CCL20 cascade	A novel therapeutic strategy for advanced CTCL [39]
miR-150	Downregulated in advanced CTCL patients	Inhibited the metastasis of CTCL cells	Inhibited the IL-22 activation in turn inhibited continuous CCL20-CCR6 interaction in CTCL cells	A key target for the treatment of advanced CTCL [40]
miR-150	Downregulated in advanced CTCL patients	Inhibited the metastasis of CTCL cells	Inhibited the migration capabilities of CTCL cells via repressing CCR6 expression	A essential therapeutic target of pan-HDACIs in advanced CTCL with metastatic potential [41]
miR-155	Upregulated in MyLa and MJ cells	Inhibited the apoptosis of MF cells	Interrupted activation of the G2/M checkpoint and decreased apoptosis	A novel therapeutic modalities for MF [30]
miR-21/miR-214/miR-486	Upregulated in SS patients and CTCL cells	Inhibited the apoptosis of CTCL cells	–	The novel diagnostic/prognostic biomarkers for CTCL [46]
miR-342	Downregulated in SS patients	Promoted the apoptosis of SS cells	–	The new possibilities for the diagnosis and treatment of SS [25]
miR-17-5p	Downregulated in SS patients	Promoted the apoptosis of SS cells	–	The new possibilities for the diagnosis and treatment of SS [25]
miR-16	Downregulated in MF patients and CTCL cells	Promoted the apoptosis of CTCL cells	Enhanced p21 expression via downregulation of the polycomb group protein Bmi1, thereby inducing cellular apoptosis	A novel therapeutic biomarkers for SAHA in the treatment of CTCL [48]
MALAT1	Upregulated in CTCL patients	Promoted the EMT and cancer stem cell phenotype	Sponged miR-124 and enhanced circulating levels of IL-6, IL-8, IL-10, TGFβ, PGE2, and MMP7, which were released by TAMs in the TME.	The potential therapeutic targets for CTCL [19]
miR-146a/miR-21	Upregulated in LCT-MF patients	Promoted the transformed phenotype in MF	Drove an immunosuppressive TME	The potential therapeutic targets for LCT-MF [50]
miR-708	Downregulated in LCT-MF patients	Inhibited the transformed phenotype in MF	Reversed an immunosuppressive TME	A potential therapeutic target for LCT-MF [50]
miR-155-5p/miR-130b-3p/miR-21-3p	Upregulated in CTCL patients, and MyLa and Hut78 cells	Inhibited CD8(+) T cell-mediated cytotoxic activity	Regulated IC expression through the JAK/STAT signaling pathway	A basis for developing synthetic anti-miRNAs to target the TME in CTCL [33]
miR-181	Upregulated in transformed CD4(+) T-cell lines and CD4(+) T-cells from SS patients	Promoted the proliferation of SS cells	Downregulated the SAMHD1 expression	Elucidating the potential role of SAMHD1 in SS pathogenesis [53]
miR-155	Upregulated in MF patients	Drove the loss of STAT4 expression and associated switch to Th2 phenotype during MF progression	The upregulation of STAT5 drove the expression of the miR-155 oncogene, which targeted STAT4 and contributed to a switch from the Th1 to Th2 phenotype	The potential therapeutic targets for CTCL [57]

*NcRNAs* non-coding RNAs, *CTCL* cutaneous T-cell lymphoma, *LncRNA* long non-coding RNA, *MiRNA* microRNA, *MF* mycosis fungoides, *SS* Sézary syndrome, *NPBMC* normal peripheral blood mononuclear cell, *HDACI* histone deacetylase inhibitor, *TAM* tumor-associated macrophage, TME tumor microenvironment, *LCT-MF* large cell transformation of mycosis fungoides, *IC* Immune checkpoint.

### NcRNAs in CTCL growth

The TMEM family consists of transmembrane proteins that span both intracellular and extracellular environments. TMEM244, a lncRNA, has been identified as a diagnostic marker for SS, a rare CTCL. It is essential for the growth of CTCL cells [14]. MALAT1 was discovered to be elevated in CTCL patients compared to healthy individuals. This upregulation induced epithelial-mesenchymal transition (EMT) and a cancer stem cell phenotype, which was enhanced by MALAT1 sponging miR-124 [19]. The p21 protein not only induces senescence and inhibits the cell cycle but also functions as a tumor suppressor. Additionally, it regulates other cellular processes such as cell migration, actin cytoskeleton remodeling, DNA repair, and apoptosis [20]. According to a recent study, miR-106b promoted the proliferation of MF tumor cells by repressing the tumor suppressors p21 and TXNIP [21]. In MF lesions, miR-93 was upregulated and could repress the cell cycle inhibitor p21, promoting the growth of malignant T cells [22]. Overexpression of miR-135a mimics in the Hut78 cells lowered GATA-3 and TOX protein levels, which subsequently inhibited cell proliferation [23]. McGirt et al. reported that miR-223 was downregulated in early-stage MF skin and could inhibit the growth of CTCL cells by targeting E2F1, MEF2C, and TOX [24]. The miR-17-5p was identified to be downregulated in SS patients and SeAx cells and could increase apoptosis and decrease cell proliferation in SS cells [25]. Lesional skin from MF patients had lower miR-195-5p expression than non-lesional MF skin or skin from healthy participants. It was suggested that miR-195-5p might operate as a tumor suppressor in MF by downregulating ARL2 and suppressing cycle arrest [26].

The miR-155 has been demonstrated to function as an oncogene in lymphomas and a variety of solid tumors [27]. Kopp et al. used the miR-155 probe to reveal that miR-155 was expressed in both malignant and non-malignant T cells in situ in CTCL, indicating that miR-155 expression varied among malignant T cells [28]. Moyal et al. investigated the expression of miR-155 in inflammatory skin conditions, early-stage MF, and tumor-stage MF. According to the study, miR-155 contributed to the development of MF into advanced stages even though it was unable to distinguish MF from benign conditions on its own [29]. In 2017, Moyal et al. also demonstrated that oncogenic miR-155 contributed to the malignant phenotype of CTCL cells by inhibiting the activation of the G2/M checkpoint in response to SL111 [30]. The JAK/STAT pathway has been reported to be constitutively activated in CTCL [31]. Fredholm et al. discovered that the JAK3/STAT5/miR-155 pathway contributed to the development of CTCL [32]. Previous research had demonstrated that miR-21-3p, miR-130b-3p, and miR-155-5p were elevated in CTCL cells and patients. In lesional skin samples, these miRNAs were found to be associated with immune checkpoint gene expression. The downregulation of miR-21-3p, miR-130b-3p, and miR-155-5p via the IL6/JAK/STAT signaling pathway in CTCL cell lines resulted in decreased CTCL cell proliferation and increased CD8 (+) T cell-mediated cytotoxic activity [33]. Cobomarsen, an inhibitor of miR-155, was created and tested by Seto et al. They also showed that miR-155 regulated several parallel survival pathways (JAK/STAT, MAPK/ERK, and PI3K/AKT), which had previously been linked to MF pathogenesis, and cobomarsen could suppress these processes in vitro [34]. As a potential target for treatment in CTCL, miR-155 was also shown to stimulate the growth of malignant T cells through the STAT5/BIC/miR-155 pathway [35].

### NcRNAs in CTCL metastasis

Metastasis is a complicated process that includes the spreading of tumor cells from their initial location, and transvascular migration to distant organs [36]. The role of MALAT1, a lncRNA, in cancer progression is not yet fully understood. However, Hong et al. found that MALAT1 expression was specifically enhanced in MF tissues. CCL21 not only mediated migration but also enhanced MALAT1 and mTOR activation in MyLa cells, resulting in cell migration [37]. The quantity of extracellular vesicles (EVs), particularly exosomes, released by cancer cells is significantly greater than that of normal cells. The study found that exosomes derived from MF cells promoted the metastasis of both MF cells and normal peripheral blood mononuclear cells (NPBMCs). These exosomes were found to be enriched with miR-1246 and miR-155, which could be targeted for new therapies and non-invasive biomarkers for MF [38]. Additionally, Matsuda et al. confirmed that miR-26 was a tumor suppressor that was linked to advanced CTCL invasion and metastasis via modulating the IL-22-STAT3-CCL20 cascade. Thus, miR-26 and its target, IL-22, are essential therapeutic targets for advanced CTCL [39].

MiR-150 has been identified as a key miRNA involved in CTCL metastasis. Ito et al. discovered that miR-150 was downregulated in advanced CTCL individuals. They revealed that miR-150 inhibited the metastasis of CTCL cells by suppressing IL-22 activation, which in turn inhibited sustained interaction of CCL20-CCR6 in CTCL cells [40]. Histone deacetylase inhibitors (HDACIs) can reinstate tumor-suppressive miRNAs in advanced CTCL. Abe et al. demonstrated that the migration of CTCL cells was inhibited and CCR6 was downregulated by pan-HDACIs, vorinostat, and panobinostat. The authors of the study found that miR-150 declined in advanced CTCL primary cases but not in the early stages. They also found that miR-150 most effectively reduced the migratory ability of advanced CTCL cells by repressing CCR6 expression [41].

### NcRNAs in the PCD of CTCL

PCD is the autonomous and orderly death of cells under genetic regulation to maintain a stable intracellular environment. Currently, known PCD types include autophagy, apoptosis, necroptosis, pyroptosis, ferroptosis, cuproptosis, and anoikis [42]. In recent years, an increasing number of studies have begun to investigate the regulatory role of PCD in the development of CTCL. TTI-621 exhibited a synergistic effect with anti-PD-L1 in reprogramming macrophages into M1-like phenotypes and suppressing CTCL cell growth. These effects were facilitated through pathways related to cell death, including apoptosis, necroptosis, and autophagy [43]. Curcumin exerts anti-cancer activity through scavenging or producing reactive oxygen species (ROS). According to Khan et al., curcumin caused apoptosis in Hut78 cells via rapidly generating ROS and regulating various cell survival and cell death pathways. Curcumin also degraded beclin-1, leading to the increase of microtubule-associated protein-I light chain 3 (LC3-I), which was a marker specific to autophagy [44]. Yosifov et al. evaluated the effects of two alkylphosphocholines (APCs) and the polyphenolic compound curcumin in vitro. The results showed that all tested drugs induced apoptosis and APCs enhanced the autophagic marker LC3B in MJ and Hut78 cells. Additionally, Co-treatment with autophagy regulators revealed that the cytotoxicity of APCs in CTCL cells was at least partly mediated by the stimulation of autophagy [45]. At present, there has been an increasing focus on PCD in CTCL. However, most studies have only explored its relationship with apoptosis. Therefore, this review will primarily examine the role of ncRNAs in CTCL apoptosis.

In recent years, several studies have reported that ncRNAs can enhance the anti-apoptotic activity of CTCL cells and promote tumor progression. In CTCL cells, miR-155 was identified to be upregulated, contributing to the malignant phenotype. It was also discovered that miR-155 could reduce apoptosis in response to SL111 and SAHA, therefore promoting MF progression [30]. SS is a leukemic subtype of CTCL that is currently incurable. Its pathogenesis is still unknown. Narducci et al. investigated the expression of 470 miRNAs in 22 SS patients and discovered 45 miRNAs that were differentially expressed between SS patients and control individuals. Among these, miR-21, miR-214, and miR-486 were upregulated in SS patients and CTCL cells and were found to contribute to CTCL cells’ apoptotic resistance [46]. Furthermore, IL-21 could induce the expression of miR-21 in CD4 (+) T cells through STAT3 activation. The level of miR-21 expression was significantly higher in CD4 (+) tumor cells found in the peripheral blood of SS patients. Inhibition of miR-21 resulted in SS cell apoptosis [47].

On the other hand, some ncRNAs may accelerate the apoptosis of CTCL cells and serve as novel therapeutic biomarkers. In the patients diagnosed with SS, miR-17-5p and miR-342 were downregulated, which could promote the apoptosis of SS cells [25]. Combating cellular senescence is an early stage in the development of cancer. Kitadate et al. demonstrated that miR-16 could cause cellular senescence in CTCL cells and was downregulated in both CTCL cells and MF patients. In CTCL cells that expressed wild-type p53, upregulated miR-16 expression could increase p21 expression by downregulating the polycomb group protein Bmi1, producing cellular senescence. In contrast, in CTCL cells that lacked functioning p53, miR-16 caused compensatory apoptosis [48].

## NcRNAs in immunological regulation

There is substantial evidence that ncRNAs play a role in the development of both local and systemic immunosuppression. This may hinder the effectiveness of immunological and therapeutic agents in patients with CTCL, potentially enabling tumorigenesis and progression by evading anti-tumor immune responses [49]. It is critical to decipher the mechanisms of ncRNA-mediated immunosuppression to restore immunosuppressed cell function.

**Fig. 3:**
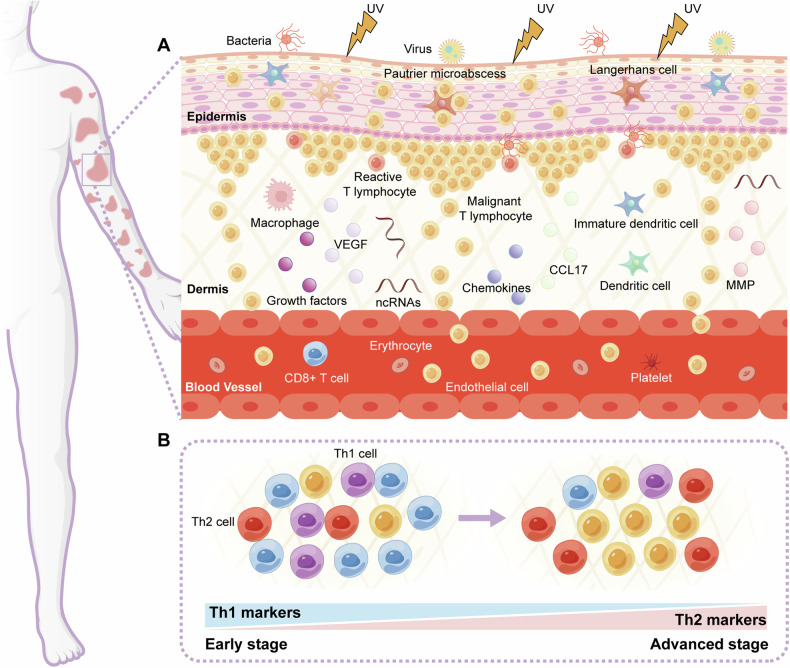
NcRNAs and multiple cell types in the TME of CTCL. **A** There are many cell types in the TME of CTCL, which can be broadly divided into three parts: (1) tumor cells: malignant CD4 (+) T cells; (2) immune cells: CD8 (+) T cells, dendritic cells, and macrophages; (3) stromal cells: keratinocytes, fibroblasts, and endothelial cells. Skin cells undergo dynamic interactions with the external environment (ultraviolet radiation, bacteria, viruses) and the internal environment (cell-cell and cell-extracellular matrix), which are altered during the pathogenesis of CTCL. Prolonged antigen presentation can lead to the formation of pautrier microabscesses in the epidermis and infiltration of CD4 (+) T lymphocytes in the epidermis and dermis. Subsequent changes lead to alterations in the skin microenvironment and T-cell activity. **B** In the early stages of the disease, CTCL skin lesions typically contain few malignant T cells among the densely infiltrated benign immune cells. A significant proportion of the benign immune cells are reactive Th1 cells and cytotoxic CD8 T cells. However, as the disease progresses, the number of infiltrating Th1 and CD8 T cells decreases. Conversely, malignant T cells accumulate and levels of Th2-associated markers increase, ultimately leading to a Th2-dominated inflammatory milieu in advanced stages of the disease. NcRNAs non-coding RNAs, TME tumor microenvironment, CTCL cutaneous T-cell lymphoma, Th T helper.

In CTCL, malignant CD4 (+) T cells interact with various components such as CD8 (+) T cells, dendritic cells, macrophages, as well as ncRNAs, and other critical actors, to form the tumor microenvironment (TME). The TME, in turn, directly and indirectly modulates tumor immunity and may be involved in the progression of CTCL (Fig. 3A). A screening of lncRNA MALAT1 in patients with CTCL revealed elevated levels compared to healthy individuals. MALAT1 was found to sponge miR-124 and enhance circulating levels of IL-6, IL-8, IL-10, MMP7, PGE2, and TGF-β, which were secreted by tumor-associated macrophages (TAMs) in the TME. This promoted the EMT and the phenotype of cancer stem cells while accelerating cell proliferation [19]. Raimondo et al. used RNA-seq analysis to investigate the expression profiles of miRNAs and mRNAs in lesional skin samples from individuals with large cell transformation of MF (LCT-MF) and non-LCT MF. It was discovered that miR-21 and miR-146a were significantly upregulated in LCT-MF, and miR-708 was the most significantly downregulated miRNA. Further analysis revealed an immunosuppressive TME in LCT-MF. The upregulation of miR-146a and miR-21, along with the downregulation of miR-708, contributed to the immunosuppressive TME, which might lead to phenotypic transformation in MF and affect tumor development [50]. Folliculotropic MF (FMF) is the most prevalent subtype of MF. MiR-155 expression was substantially higher in biopsies from tumor-stage FMF than in early-stage FMF and inflammatory dermatoses. Additionally, CD68 (+) macrophages, CD20 (+) B cells, and dermal Ki-67 (+) proliferating lymphocytes were significantly increased in tumor-stage FMF compared to early-stage FMF [51].

In the TME of CTCL, immune cells, including malignant CD4 (+) T cells, CD8 (+) T cells, and TAMs, interact to create an environment that promote CTCL growth [5]. Immune checkpoints (ICs) also play an important role in immunological depletion and disease development. In CTCL skin samples, miR-21-3p, miR-130b-3p, and miR-155-5p were found to have a positive correlation with IC gene expression. These miRNAs were also discovered to regulate IC expression in CTCL cells via the JAK/STAT signaling pathway. Downregulating miR-21, miR-130, and miR-155 resulted in reducing CTCL cell proliferation and increasing CD8 (+) T cell-mediated cytotoxic activity. These findings provide light on the process of miRNA-induced T cell exhaustion, which can be used as a foundation for creating synthetic anti-miRNAs to target the TME in CTCL [33]. SS is a rare variant of CTCL distinguished by the aggressive dissemination of neoplastic CD4 (+) T-cells (Sézary cells) from the skin into the circulation, with subsequent metastasis to visceral organs. The expression of miR-21 was higher in SS cells than in CD4 (+) T cells from healthy individuals, which was regulated by STAT3. Silencing miR-21 could increase apoptosis, indicating that miR-21 played a functional role in the leukemogenic development [47]. SAMHD1’s unique role as a dNTPase puts it at the intersection of cell proliferation, cellular cycle modulation, and mutagenesis, making it a potential tumor suppressor [52]. In SS cells, the expression of SAMHD1 was downregulated via miR-181 compared with normal CD4 (+) T-cells. MiR-181 was a significant modulator of SAMHD1 protein expression in SS cells, most likely via translational suppression [53].

The TME of MF and SS exhibit characteristics of a T helper (Th) 2 phenotype, which may suppress immune responses related to tumors [54] (Fig. 3B). The pathogenesis of CTCL has been linked to the deregulation of STAT signaling. Recent reports indicate that malignant cells acquire a Th2 cell phenotype due to the loss of STAT4 expression [55]. Litvinov et al. demonstrated that in CTCL, the genes STAT4 and STAT6 were inversely regulated. STAT4 expression correlated with the Th1 phenotype, whereas STAT6 was linked to the Th2 phenotype. The upregulation of oncogenic miR-155 might drive the loss of STAT4 expression and the related transition to the Th2 phenotype during MF development [56]. Similarly, IL-2, IL-7, and IL-15 could activate STAT5 via JAK1 and JAK3 kinases during the early stage of CTCL. This upregulation of STAT5 drove the miR-155 expression, which targeted STAT4 and contributed to a transition from the Th1 to Th2 phenotype. In later stages, STAT3 activation enhanced survival and resistance to apoptosis, potentially promoting carcinogenesis [57].

## NcRNAs as promising diagnostic biomarkers

CTCL is a group of malignant tumors with diverse histological, clinical, and prognostic features, classified based on their impact on the skin. CTCL is characterized by the presence of malignant T cells, which proliferate in a chronic inflammatory milieu and expand into the skin. SS and MF are the two invasive subtypes of CTCL and are the most commonly occurring [58]. Diagnosing CTCL can be challenging as it presents clinical and pathological similarities to benign tumors. Furthermore, CTCL is often diagnosed at an advanced stage because of its slow progression, resulting in a dismal prognosis. The ncRNA expression in CTCL has been suggested as a diagnostic target for cancer (Table 2). Previous studies have successfully found ncRNA signatures to distinguish CTCL from other comparable illnesses and cancers, such as benign inflammatory disorders (BIDs) [59]. These particular ncRNA signatures have the potential to differentiate CTCL from other tumors/lymphomas, as well as distinguish between distinct variants of CTCL, including MF, SS, FMF, transformed MF (TMF), and erythrodermic MF (eMF) [60].

**Table 2:** The value of ncRNAs in CTCL diagnosis.

CTCL subtypes	NcRNAs	Dysregulation	Significance
CTCL	TMEM244	Upregulated in HH and SeAx cells	Differentiated CTCLs from other malignancies [14]
MF	miR-34a/miR-29a	Upregulated in patients with tumor stage MF	Drove the pathogenesis of MF [62]
MF	miR-155/miR-1246	Upregulated in exosomes isolated from CTCL cells and MF patients	Associated with more advanced lesions of MF and served as promising noninvasive biomarkers for MF [38]
CTCL	miR-155/miR-203/miR-205	Upregulated miR-155 and downregulated miR-203/miR-205 in the plasma of CTCL patients	Diagnosed CTCLs from benign lesions [63]
CTCL	miR-130b/miR-142-3p/miR-155/miR-200b/miR-203	Upregulated miR-130b/miR-142-3p/miR-155 and downregulated miR-200b/miR-203 in CTCL patients	Diagnosed CTCLs from BIDs [64]
MF	miR-155/miR-146a/miR-146b-5p/miR-342-3p/let-7i*/miR-203/miR-205	Upregulated miR-155/miR-146a/miR-146b-5p/miR-342-3p/let-7i* and downregulated miR-203/miR-205 in early MF patients	Diagnosed early MF from AD [66]
MF	miR-155/miR-92a/miR-93	Upregulated in patients with tumor stage MF	Diagnosed tumor stage MF from eczema and lichen planus [67]
MF	miR-93	Downregulated in MF patients	Diagnosed MF from eczema [68]
MF	miR-142-3p/miR-150/miR-146b	Upregulated in early-stage MF patients	Diagnosed early-stage MF from psoriasis [60]
MF	miR-26a/miR-222/miR-181a/miR-146a	Upregulated in early-stage MF patients	Diagnosed early-stage MF from BIDs [69]
MF	miR-17~92	Upregulated in unilesional MF patients	Diagnosed unilesional MF from early-stage MF and BIDs [70]
MF	miR-181a/miR-146a	Upregulated in advanced MF patients	Improved both diagnosis and risk prediction [69]
CTCL	miR-17/miR-92/miR-106b/miR-25/miR-106a/miR-363	Upregulated in more advanced CTCL patients	Improved both diagnosis and risk prediction [66]
MF	miR-16	Upregulated in advanced MF patients	Predicted an aggressive course of MF [68]
SS/eMF	TMEM244	Upregulated in SS patients	Diagnosed SS from eMF [71]
SS/eMF	miR-373-3p/miR-509-3p/miR-491-5p/miR-340-5p/miR-370-3p/miR-127-5p/miR-193a-3p/miR-409-3p/miR-495-3p/miR-410-3p/miR-323a-3p/miR-539-5p/miR-95-3p	Downregulated in SS patients	Diagnosed SS from eMF [72]
SS/eMF	miR-425-5p/miR-20a-5p/miR-126-5p/miR-106b-5p/miR-19a-3p/miR-21-5p/miR-142-5p/miR-181a-3p/miR-155-5p/miR-215-5p/miR-7-5p/miR-142-3p/miR-146a-5p/miR-625-3p	Upregulated in SS patients	Diagnosed SS from eMF [72]
C-ALCL	miR-155/miR-27b/miR-30c/miR-29b	Upregulated in PCALCL patients	Diagnosed PCALCL from BIDs [73]
C-ALCL/tumor-stage MF	miR-155/miR-27b/miR-93/miR-29b/miR-92a	Upregulated in PCALCL patients	Diagnosed PCALCL from tumor-stage MF [73]
FMF	miR-155	Upregulated in tumor-stage FMF compared with early-stage FMF and BIDs	Diagnosed FMF from BIDs and predicted an aggressive course of FMF [51]
FMF/TMF	miR-93-5p/miR-181a/miR-34a	Upregulated in FMF and TMF patients compared to controls	Served as promising noninvasive biomarkers for FMF and TMF [78]
FMF/TMF	miR-155/miR-223	Upregulated in FMF patients	Diagnosed FMF from TMF [78]
FMF/TMF	miR-181b/miR-326	Downregulated in FMF patients	Diagnosed FMF from TMF [78]
FMF/TMF/tumor-stage MF	miR-17/miR-18a	Downregulated in FMF and TMF compared to tumor-stage MF	Diagnosed FMF and TMF from tumor-stage MF, and predicted an aggressive course of MF [79]
FMF/TMF/tumor-stage MF	miR-19b/miR-92a/miR-155	Upregulated in FMF and TMF compared to tumoral MF	Diagnosed FMF and TMF from tumor-stage MF, and predicted an aggressive course of MF [79]
Unilesional MF/early MF	miR-17~92 members	Upregulated in unilesional MF patients	Diagnosed unilesional MF from early MF and BIDs [70]
MF	miR-106b-5p/miR-148a-3p/miR-338-3p	Upregulated miR-106b-5p and downregulated miR-148a-3p/miR-338-3p in early-stage MF patients	Separated patients into high-risk and low-risk groups of disease progression [80]
MF	miR-106b	Upregulated in MF patients	Exerted the prognostic role in progression of MF [21]
CTCL	miR-155	Upregulated in CTCL patients	Predicted an aggressive course of CTCL [28]
MF	miR-17/miR-19b	Upregulated in MF patients with genomic alterations	Predicted a bad response to treatment and short survival [79]
CTCL	miR-155	Upregulated in CTCL patients	Associated with poor prognosis [64]
CTCL	miR-200b	Downregulated in CTCL patients	Associated with good prognosis [64]

*NcRNAs* non-coding RNAs, *CTCL* cutaneous T-cell lymphoma, *MF* mycosis fungoides, *MiRNA* microRNA, BID Benign inflammatory disorder, *AD* atopic dermatitis, *SS* Sézary syndrome, *eMF* erythrodermic mycosis fungoides, *PCALCL* primary cutaneous anaplastic large cell lymphoma, *FMF* folliculotropic mycosis fungoides, *TMF* transformed MF.

### NcRNAs in diagnosing CTCL

As a type of ncRNAs, lncRNAs are dysregulated in a variety of human cancers, including CTCL. In a study by Lee et al., the SS transcriptome was characterized, and 12 differentially expressed lncRNAs were identified [61]. TMEM244 was found to be upregulated in HH and SeAx cells and might be used as a CTCL diagnostic marker [14]. Multiple miRNAs have been linked to CTCL, not only for diagnostic purposes but also as oncogenes that drive CTCL pathogenesis. Papadavid et al. revealed that miR-34a was an oncogenic molecule whereas miR-29a was a tumor suppressor, emphasizing their significance in the molecular pathogenesis of tumor-stage MF [62]. MiR-155 is the most representative of these miRNAs, and its overexpression has been frequently found in CTCL-related research [33]. Exosomes isolated from CTCL cells and MF patients showed elevation of miR-1246 and miR-155, which might promote cell motility. These miRNAs might be useful non-invasive biomarkers for MF, as they were related to more advanced lesions of the disease [38].

Individual miRNAs may not have independent diagnostic significance in CTCL, however, certain combinations of miRNAs can be effective. For example, CTCL could be diagnosed from benign lesions with 100% specificity and 94% sensitivity using the plasma miRNA classifier containing elevated miR-155 together with decreased miR-203/miR-205 [63]. Shen et al. constructed and validated a diagnostic classifier for CTCL that included miR-155, miR-203, miR-130b, miR-200b, and miR-142-3p. This classifier was able to discriminate CTCL from BIDs [64]. Additionally, miRNA profiling revealed considerable overexpression of miR-711, miR-326, and miR-663, and suppression of miR-718, miR-205, and miR-203 in CTCL. A quantitative real-time polymerase chain reaction (qRT-PCR)-based classifier consisting of miR-205, miR-203, and miR-155, could differentiate CTCL from benign diseases. The classifier demonstrated the high diagnostic potential of miRNAs in CTCL with a classification accuracy of 95% [65].

Early lesions of MF present as plaques and patches, and can mimic inflammatory skin conditions, which can make it challenging to differentiate from BIDs like atopic dermatitis, chronic eczema, and psoriasis. A recent observation identified differentially expressed miRNAs in early lesions of MF and atopic dermatitis (AD). The miRNAs that could differentiate early MF from AD comprised both elevated (miR-342-3p, miR-155, let-7i*, miR-146b-5p, miR-146a) and decreased (miR-205, miR-203) miRNAs [66]. The miRNA expression profiling showed that miR-93, miR-92a, and miR-155 were elevated in tumor-stage MF compared to BIDs (eczema and lichen planus) [67]. It is important to note that the breadth of application of miR-93 may vary. Talaat et al. identified miR-93 as being downregulated in MF cases compared to eczema cases [68]. Early-stage MF was found to have miRNA characteristics that overlapped with those of psoriasis using a qRT-PCR platform for miRNAs. However, 39 miRNAs were specific for MF, including miR-150, miR-146b, and miR-142-3p [60]. Furthermore, previous studies had indicated that a limited set of miRNAs (miR-146a, miR-26a, miR-181a, and miR-222) might be adequate in distinguishing MF from BIDs [69]. Additionally, unilesional MF had a distinct miRNA profile compared to normal early MF, with increased expression of miR-17~92 families [70].

Early-stage MF can be indolent, but some patients may develop rapidly to terminal stages. Observing the progression of MF will be advantageous in its management. In a separate study, miR-146a and miR-181a were shown to be markedly dysregulated in early and terminal MF stages [69]. When contrasting early-stage MF to more terminal CTCL, Ralfkiaer et al. found that the carcinogenic miR-106a/363, miR-106b/25, and miR-17/92 clusters were significantly upregulated. In 16 patients with available follow-up data, 72 miRNAs were found to be differentially expressed between aggressive and non-aggressive illness [66]. The study reported that miR-16 was considerably elevated in terminal cases of MF in contrast to early-stage cases. This might be utilized to anticipate an aggressive progression of MF [68]. These findings suggest that miRNA profiling in CTCL can improve both diagnosis and risk prediction.

### NcRNAs in distinguishing CTCL subtypes

The differential diagnosis of CTCL subtypes is crucial due to the many subtypes that exist. NcRNAs have been reported to be useful in this regard, as they are involved in the progression of CTCL and can aid in distinguishing between subtypes. Distinguishing between eMF and SS can be challenging because of their comparable clinical and histological characteristics. According to a study, the expression of lncRNA TMEM244 was discovered to be elevated in SS in contrast to eMF and healthy individuals, indicating its potential as a diagnostic tool [71]. Additionally, distinct miRNA expression profiles were observed between the two diseases, with 14 miRNAs with lower expression levels and 13 miRNAs with higher expression levels in eMF in contrast to SS [72]. Primary cutaneous anaplastic large cell lymphoma (PCALCL) is characterized by large cells expressing the CD30 antigen in over 75% of tumor cells. Benner et al. used miRNA-Q-PCR to validate the overexpression of miR-27b, miR-29b, miR-30c, and miR-155 in PCALCL versus BIDs. The miRNA-Q-PCR showed a considerable upregulation of miR-29b, miR-27b, miR-92a, miR-93, and miR-155 in PCALCL in comparison to MF at the tumor stage [73].

Based on its clinicopathological features, MF can be classified into several subtypes [74]. FMF is an uncommon subtype of MF that has particular histopathological characteristics, where tumor cells surround and invade the follicular epithelium, with mild or no epidermotropism [75]. At present, there is no standardized treatment for FMF [76]. In addition, a small proportion of individuals with MF may experience the transformation of large cells, also known as TMF, which is diagnosed by the existence of over 25% large cells on biopsy of an MF lesion [77]. Nevertheless, the molecular basis of both FMF and TMF is not yet completely understood. Certain miRNAs have been implicated in the etiology and development of classical MF and can assist in the differential diagnosis of FMF. There are two distinguishable clinicopathological stages of FMF: the early inert stage and the tumor stage. According to a study, miR-155 expression in biopsies of FMF in the tumor stage was significantly higher than in early FMF and inflammatory skin disease [51]. An analysis of the expression of 11 miRNAs in 9 cases of FMF and 7 cases of TMF identified miR-34a, miR-181a, and miR-93-5p as substantially elevated in both FMF and TMF. Meanwhile, the study found that miR-223 and miR-155 were overexpressed in FMF, whereas miR-326 and miR-181b were overexpressed in TMF cases compared to controls [78]. Similarly, Garaicoa et al. investigated skin samples from 36 patients, including 16 tumor-stage MF, 13 TMF, and 7 FMF, and observed higher expression of miR-18a and miR-17 were highly expressed in tumoral MF, while miR-155, miR-92a, and miR-19b were overexpressed in TMF and FMF [79]. Unilesional MF, featured by an isolated lesion, is clinically and pathologically indistinguishable from the multifocal patch or typical MF. The miRNA profile of unilamellar MF differed significantly from that of early-stage MF and inflammatory dermatoses. The former exhibited higher levels of miR-17~92 families, along with Th1 skewing [70].

### NcRNAs in CTCL prognosis

NcRNAs can improve diagnostic accuracy and predict prognosis in CTCL. Understanding the involvement of ncRNAs in the pathogenesis of CTCL can help determine the appropriate therapy for individual patients. In 2018, Lindahl et al. created a 3-miRNA classifier, which successfully distinguished between patients with high and low risk of MF development. The classifier, based on miR-338-3p, miR-148a-3p, and miR-106b-5p, provided substantial significance to current clinical prognostic markers, potentially allowing for a more tailored therapy [80]. Among these markers, miR-106b stood out as the most potent prognostic indicator of MF development. As demonstrated by cellular experiments, miR-106b could upregulate with the advancing MF stage and repress the tumor inhibitors TXNIP and cyclin-dependent kinase inhibitor p21, promoting MF tumor cell growth. This substantiated that miR-106b had a functional and prognostic impact on MF development [21].

Over the past year, miR-155 has gained increasing attention as an indicator of CTCL prognosis. Increased levels of miR-155 have been detected in skin lesions of CTCL patients. MiR-155 is implicated in disease development from early inert to the aggressive tumoral stage. Higher expression of miR-155 is linked to more severe illness [28]. Garaicoa et al. discovered that miR-17 and miR-18a were more highly expressed in tumor-stage MF than in FMF and TMF. Conversely, FMF and TMF had higher levels of miR-155, miR-92a, and miR-19b than tumor-stage MF. In addition, they detected increased expression of miR-19b and miR-17 in the genomic alteration group, which showed poor response to treatment and short survival compared to cases without alterations [79]. The study also found that miR-200b and miR-155 were significantly related to overall survival (OS) in CTCL patients but in opposite directions. The combination of miR-200b, Ki-67, and miR-155 was more accurate in predicting the 5-year OS of CTCL than Ki-67 alone. Notably, in patients with different stages of MF, miR-200b was significantly associated with OS [64].

## NcRNA enlightenments in CTCL therapeutics

Local and systemic therapies have been developed for the treatment of CTCL, which have had a positive impact on tumor burden and quality of life. However, conventional therapies still have limitations, highlighting the need for further medical advancements (Table 3). In patients with advanced MF and genomic alterations, such as increased expression of miR-17 and miR-19b, treatment response was poor and survival was short [79]. Based on current clinical trials, ncRNA-based therapy appears to be viable. STAT5 proteins might drive the expression of the carcinogenic BIC/miR-155, thereby promoting malignant T cell proliferation, making it a potential target for therapy in CTCL [35]. Extracorporeal photopheresis (ECP) is a treatment for CTCL. The mean time to achieve the clinical effect of this drug was 22.4 ± 9.6 weeks. This indicated that patients should undergo ECP treatment for up to 8 months to precisely evaluate its effectiveness. However, this therapy was not appropriate for patients with advanced CTCL [81]. After ECP treatment, PBMC miR-342, miR-223, and miR-191 were elevated at 3 months, which could anticipate clinical effects of ECP between 6 and 12 months [82]. Bacteria and other environmental factors can accelerate the development of CTCL. Compared to non-lesional or healthy skin, Staphylococcus aureus was more prevalent in skin lesions of MF patients. The presence of Staphylococcus aureus and its enterotoxins could lead to increased expression of oncogenic miR-155, which might be linked to recurrent skin infections in MF patients [83]. Given the importance of antimicrobial therapy and miRNAs in MF, combining miRNA-based therapy and antimicrobial therapy may have synergistic therapeutic benefits and improve the outcome of MF patients. This is particularly significant for advanced MF with extensive involvement.

**Table 3:** The value of ncRNAs in CTCL therapeutics.

NcRNAs	Dysregulation	Mechanism	Clinical significance
miR-17/miR-19b	Upregulated in patients with advanced MF	–	Predicted the poor treatment response and short survival [79]
miR-155	Upregulated in CTCL patients	Modulated by the STAT5/BIC/miR-155 pathway	Promoted malignant T cell proliferation and served as a potential target for therapy in CTCL [35]
miR-191/miR-223/miR-342	Upregulated in PBMC of patients with advanced CTCL at 3 months post-ECP therapy	–	Predicted the clinical response of patients with advanced CTCL to ECP between 6 and 12 months [82]
miR-155	Upregulated in secondary skin infections in patients with MF	Increased by the presence of Staphylococcus aureus and its enterotoxins	Emphasized the importance of the combination of miRNA-based therapy and antimicrobial therapy [83]
miR-155	Upregulated in MF patients	Cobomarsen reduced the expression of multiple gene pathways related to cell survival through blocking miR-155	Cobomarsen reduced cell proliferation and activated apoptosis in CTCL [34]
miR-214	Upregulated in purified CD4(+) neoplastic T cells from patients with CTCL	Regulated by the TWIST1/BRD4/miR-214 regulatory loop	Emphasized a major oncogenic pathway in CTCL that can be targeted [84]
miR-26	Downregulated in CTCL patients	Regulated the IL-22-STAT3-CCL20 cascade	Emphasized a novel approach for treating advanced CTCL by targeting IL-22 [39]
miR-150	Downregulated in advanced CTCL primary patients	Pan-HDACIs targeted miR-150 and/ or CCR6	Indicated that miR-150 and its target, CCR6, are essential therapeutic targets of pan-HDACIs in advanced CTCL with metastatic potential [41]
miR-22	Downregulated in CTCL patients	Modulated by the Jak3/STAT3/STAT5 signaling	Reversed by curcumin, a nutrient with anti-Jak3 activity and functions as an HDACIs [86]
miR-16	Downregulated in advanced CTCL primary patients	Induced senescence and promoted apoptosis in CTCL cells	SAHA reinstated the expression of miR-16 and its essential targets [48]
miR-93	Upregulated in patients with advanced MF	Suppressed cell cycle inhibitor p21 and promoted malignant T-cell proliferation	SAHA inhibited miR-93 and then partially induced p21 expression [22]
miR-122	Upregulated in patients with advanced MF	Through a signaling pathway involving Akt activation and p53 inhibition	Reduced sensitivity to chemotherapy-induced apoptosis [90]
miR-29b	Downregulated in CD4(+) T cells of CTCL patients	Resulted in BRD4 overexpression	Prevented the progression of CTCL by interfering with BRD4-mediated pathogenesis [91]

*NcRNAs* non-coding RNAs, *CTCL* cutaneous T-cell lymphoma, MiRNA microRNA, MF mycosis fungoides, *PBMC* peripheral blood mononuclear cell, *ECP* extracorporeal photopheresis, *HDACI* histone deacetylase inhibitor, SAHA suberoylanilide hydroxamic acid.

CTCL is an indication for miRNA-interfering therapies, allowing deeper insights into the epigenetic deregulation of CTCL and miRNA-regulated tumourigenesis. Due to the crucial involvement of miR-155 in MF, Seto et al. developed and tested a targeted nucleic acid-modified miR-155 oligonucleotide suppressant, cobomarsen. Cobomarsen had the potential to be used as a therapeutic agent for CTCL by inhibiting miR-155 to reduce the expression of numerous gene pathways associated with cell survival, resulting in reduced cellular growth and activated apoptosis [34]. In addition, miR-214 levels in CD4 (+) tumor T cells from CTCL patients were found to be substantially higher than those in healthy individuals. The study showed that the BET protein BRD4 and TWIST1 co-regulated miR-214 expression in CTCL cell lines and patient samples. Treatment with the JQ1, a BRD4 inhibitor, reduced miR-214 levels. Thus, the TWIST1/BRD4/miR-214 regulatory pathway was a crucial carcinogenic mechanism that could be targeted in CTCL [84].

HDACIs are clinically permitted for CTCL therapy and have previously demonstrated efficacy in routine clinical applications [85]. In advanced CTCL, HDACIs can recover cancer suppressor miRNAs including miR-26, miR-150, miR-22, and miR-16. Among these, miR-26 was found to be related to cancer invasion and metastasis in advanced CTCL through regulation of the IL-22-STAT3-CCL20 pathway. Consequently, the targeting of IL-22 might represent a novel approach to the treatment of advanced CTCL [39]. The pan-HDACIs, vorinostat, and panobinostat, had been shown to reduce CTCL metastasis by targeting miR-150 and/or CCR6, which were important therapeutic targets for advanced CTCL with metastatic potential [41]. Furthermore, the miR-22 expression was repressed in CTCL cells as a result of the dysregulated Jak3/STAT3/STAT5 pathway. This repression could be reversed by curcumin, a substance with anti-Jak3 effects and HDACIs function [86]. Suberoylanilide hydroxamic acid (SAHA), the class I and II HDACI, had been shown to accumulate acetylated histones in CTCL, leading to cell cycle arrest and apoptosis [87]. It was worth noting that the expression of miR-16 decreased in primary CTCL as it progressed from early to advanced stages. SAHA was able to reinstate miR-16 and its critical targets, inducing senescence in wild-type p53 CTCL cells and promoting apoptosis in dysfunctional p53 cells [48]. Recent studies have reported aberrant miR-93 expression in MF lesions and the association of dysregulated miR-93 expression with advanced MF. Oncogenic miR-93 suppressed cell cycle inhibitor p21 and promoted malignant T-cell proliferation. Furthermore, SAHA partially induced p21 expression by inhibiting miR-93 [22]. It was noteworthy that HDACIs upregulated STAT4 expression and simultaneously downregulated STAT6 expression in MyLa cells, which was similar to the impact of miR-155 knockdown [56].

During PCD, various lethal subroutines can impact cancer development and response to therapy. Mutations that impair the PCD pathway during the early stage of cancer initiation may render cells resistant to anti-cancer therapy. It is important to note that avoiding PCD is a characteristic of cancer. Therefore, exploring the key genes, proteins, and pathways that govern PCD is of great significance in providing new therapeutic strategies for CTCL and has promising clinical applications. Bexarotene activated the retinoid X receptor, which was a commonly used anti-cancer agent for CTCL and had been shown to promote autophagy [88]. Vorinostat, a drug approved to treat advanced primary CTCL, demonstrated a significant anti-cancer impact both in vitro and in vivo via modulating the expression of genes related to ferroptosis [89]. MiR-122 was found to be elevated in the advanced MF stages, and its overexpression lowered the susceptibility to chemotherapy-induced apoptosis through a signaling circuit involving Akt activation and p53 suppression in CTCL cells [90].

Advanced CTCL is a chemotherapy-resistant disease and represents a significant area of unmet medical need. It is critical to identify functionally important ncRNAs that regulate CTCL cell apoptosis and influence the efficacy of CTCL chemotherapy due to the known role of ncRNAs in regulating cellular signaling. MiR-122 was expressed in cancerous T-cell infiltration and was elevated in advanced MF. The overexpression of miR-122 surprisingly reduced susceptibility to chemotherapy-induced death through Akt-activated and p53-inhibited signaling pathways [90]. Decreased miR-29b levels in CTCL patients’ CD4 (+) T cells resulted in BRD4 overexpression, which was associated with elevated levels of the interleukin-15 (IL-15) receptor complex and tumor-related genes (including RBPJ and NOTCH1). Furthermore, interfering with BRD4-mediated pathogenesis prevented the progression of CTCL. This was achieved by either recovering the expression of miR-29b through bortezomib medication or by direct inhibition of BRD4 binding through JQ1 therapy [91].

## Conclusion

**Fig. 4:**
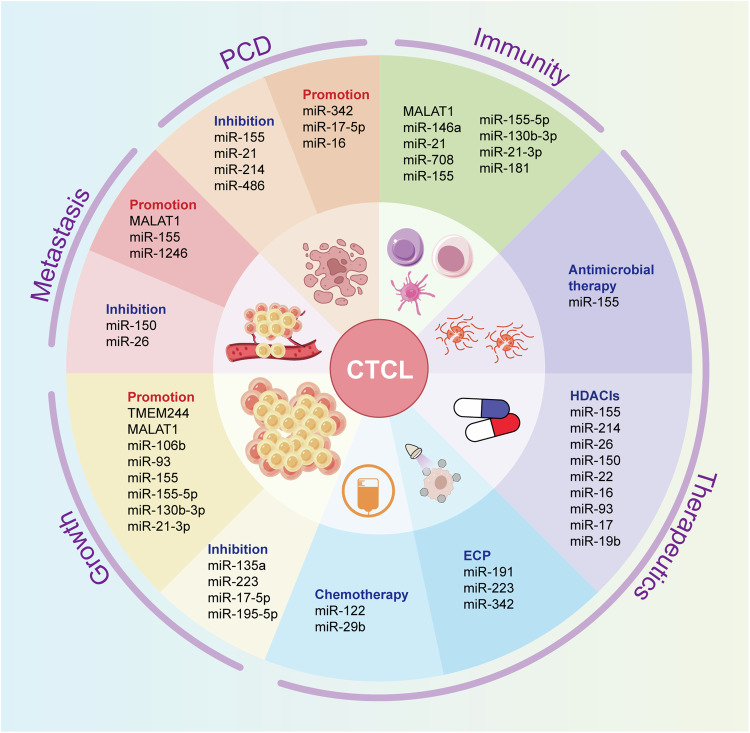
The biological function of ncRNAs in CTCL. NcRNAs play a key role in cell communication, thus mediating a wide range of CTCL processes and having a confounding effect on CTCL progression. More importantly, ncRNAs can be taken up non-randomly by heterologous and homologous cells, affecting post-transcriptional gene regulation and leading to behavioral changes characterized by CTCL growth and metastasis, PCD, immunological regulation, and treatment resistance. *NcRNAs* non-coding RNAs, *CTCL* cutaneous T-cell lymphoma, *PCD* programmed cell death.

CTCL is a form of T-cell lymphoma affecting the skin. Although each subtype has a distinct molecular and disease progression profile, they share overlapping physical manifestations. The pathogenesis and spread of the disease involves complicated connections between the innate and adaptive immune systems, keratinocytes, and the skin microbiota, cancerous and normal T cells. It is therefore challenging to accurately predict treatment response and survival. Although imaging modalities including PET and CT scans are effective in diagnosing the location, staging, and dissemination of tumors, they are unable to provide helpful guidance on the best treatment options with the least damage and the longest survival. In addition, early-stage CTCL often presents with similar clinical features to BIDs, which can make it difficult to distinguish between the two. As a result, opportunities for early intervention may be missed, with the consequence that later-stage disease may be more devastating. Disease-specific molecular indicators in cancer tissue and circulatory fluids show promise for molecular diagnosis, potentially decreasing tumor mortality by indicating optimal therapies to improve survival. NcRNAs have been discovered as key actors in CTCL pathogenesis and are associated with its progression, diagnosis, and treatment (Fig. 4). Several researchers have implicated them as promising diagnostic and therapeutic indicators for CTCL. This discovery presents a fresh avenue for diagnosing, treating, and predicting the outcome of CTCL by identifying these novel gene-modulating targets. However, aberrant ncRNAs have also been found in various inflammatory diseases and cancers. While some studies have suggested that diagnostic confidence can be improved by using classifiers/signatures of multiple miRNA associations, the use of disease-specific ncRNAs remains a challenging issue. The function of some ncRNAs in CTCL is still controversial. Further studies and confirmation in larger cohorts are required. In addition, ncRNA-based therapies for CTCL are still in their infancy. Delivering ncRNAs reliably and specifically to the site of action is a significant challenge in the application of ncRNA-based therapies. Further research could investigate the regulatory mechanisms of exosomal ncRNAs in CTCL progression, as well as the correlation between ncRNAs and different kinds of PCD in CTCL. This may provide new insights into innovative diagnostic and therapeutic approaches for CTCL.
